# Nursing Staffing Is Associated With Mobility and Food Intake in Older Hospitalized Patients

**DOI:** 10.1093/geroni/igaa057.453

**Published:** 2020-12-16

**Authors:** Orly Tonkikh, Efrat Shadmi, Anna Zisberg

**Affiliations:** 1 University of Haifa, Haifa, Israel; 2 Faculty of Social Welfare and Health Sciences, University of Haifa, Haifa, Israel, Haifa, Isreal, United States

## Abstract

Hospitalization processes related to patient mobility and food-intake significantly affect outcomes of older adults. Nurses are the front-line personnel responsible for promoting performance of these functioning-preserving processes. The degree to which nursing skill-mix is related to their performance is unclear. We investigated the association between staffing and hospitalization processes in a cohort of 836 older adults aged 70+ admitted to internal units for non-disabling conditions. Mobility and food-intake were assessed within 2 days of admission using validated questionnaires. Nurse-patient ratios and nursing skill-mix (i.e. registered nurses, nurse aides, and advanced practice nurses) were assessed using administrative and payroll/roster data. Decision-trees were developed for mobility and food-intake applying classification and regression tree analysis. The mobility decision-tree identified four characteristics that subdivided the patients into eight segments (nodes) (pre-admission functioning, sex, malnutrition risk and percent of advanced practice nurses). The food-intake decision-tree identified five characteristics (pre-admission functioning, sex, chronic morbidity, age and percent of nurse aids) that subdivided the patients into ten nodes. Percent of advanced practice nurses and the percent of nurse aids classified low functioning patients: higher percent of advanced practice nurses (>30% vs. ≤30%) was associated with higher probability of walking in corridors (20.7%) versus inside the room (4.3%), and higher percent of nurse aids (>23% vs. ≤23%) was associated with higher probability of eating more than half of the served meals (83.9%) versus others (66.3%). This study shows that staffing levels are associated with better performance of functioning-preserving processes. Future studies should investigate staffing interventions improving functioning-preserving processes.

